# Detection of Severe Lung Infection on Chest Radiographs of COVID-19 Patients: Robustness of AI Models across Multi-Institutional Data

**DOI:** 10.3390/diagnostics14030341

**Published:** 2024-02-05

**Authors:** André Sobiecki, Lubomir M. Hadjiiski, Heang-Ping Chan, Ravi K. Samala, Chuan Zhou, Jadranka Stojanovska, Prachi P. Agarwal

**Affiliations:** 1 Department of Radiology, University of Michigan, Ann Arbor, MI 48109, USA; sobieckiandre@gmail.com (A.S.); chanhp@umich.edu (H.-P.C.); chuan@med.umich.edu (C.Z.); prachia@med.umich.edu (P.P.A.); 2 Office of Science and Engineering Laboratories, Center for Devices and Radiological Health, U.S. Food and Drug Administration, Silver Spring, MD 20993, USA; ravi.samala@fda.hhs.gov; 3 Department of Radiology, New York University, New York, NY 10016, USA; jadranka73@icloud.com

**Keywords:** severe lung infection, COVID-19, deep learning, diagnosis, classification

## Abstract

The diagnosis of severe COVID-19 lung infection is important because it carries a higher risk for the patient and requires prompt treatment with oxygen therapy and hospitalization while those with less severe lung infection often stay on observation. Also, severe infections are more likely to have long-standing residual changes in their lungs and may need follow-up imaging. We have developed deep learning neural network models for classifying severe vs. non-severe lung infections in COVID-19 patients on chest radiographs (CXR). A deep learning U-Net model was developed to segment the lungs. Inception-v1 and Inception-v4 models were trained for the classification of severe vs. non-severe COVID-19 infection. Four CXR datasets from multi-country and multi-institutional sources were used to develop and evaluate the models. The combined dataset consisted of 5748 cases and 6193 CXR images with physicians’ severity ratings as reference standard. The area under the receiver operating characteristic curve (AUC) was used to evaluate model performance. We studied the reproducibility of classification performance using the different combinations of training and validation data sets. We also evaluated the generalizability of the trained deep learning models using both independent internal and external test sets. The Inception-v1 based models achieved AUC ranging between 0.81 ± 0.02 and 0.84 ± 0.0, while the Inception-v4 models achieved AUC in the range of 0.85 ± 0.06 and 0.89 ± 0.01, on the independent test sets, respectively. These results demonstrate the promise of using deep learning models in differentiating COVID-19 patients with severe from non-severe lung infection on chest radiographs.

## 1. Introduction

Deep-learning-based models, commonly referred to as artificial intelligence (AI) models, have been developed to assist physicians in analyzing medical images and in making diagnostic decisions in the past decade. AI models have been developed to analyze chest radiographs (CXR) for classifying various lung diseases. For COVID-19, AI has been shown to quantify the extent of lung involvement that correlates with ICU admission, intubation, and death [1]. Reliable quantification of disease characteristics using AI models has the potential to help monitor disease progression or regression, reducing some of the healthcare burden.

**Figure 1:**
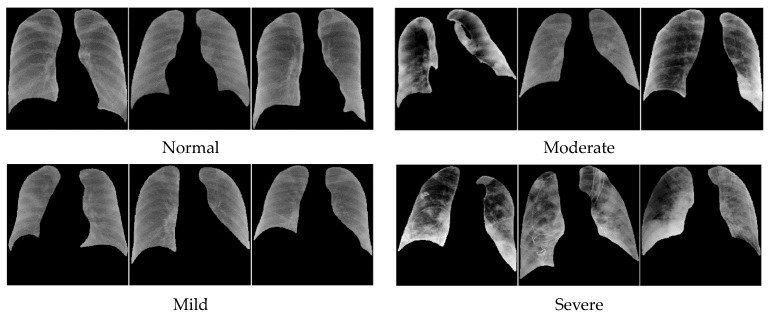
Different degrees of COVID severity.

Characterization of the level of lung infection on chest radiograph of COVID-19 patients is generally a challenging task due to the wide variation of its appearance. Nair et al. [2] conducted a study that quantified reader agreement in their diagnosis of COVID-19. In their study, there were four groups of readers: chest consultant, general consultant, specialist registrar radiologists, and infectious disease clinicians. They concluded that the reader agreement was low. AI-aided reading could potentially mitigate this issue as has been shown in other types of clinical tasks [3,4,5]. For instance, Figure 1 illustrates the different degrees of severity from COVID-19 infection: (1) normal, (2) mild, (3) moderate, and (4) severe [6,7,8]. Visually, in terms of pixel density, shape, and texture, while normal and mild may be distinguishable from those of severe cases, moderate and severe COVID-19 infections are much more similar.

Previous studies have proposed AI methods for identifying severe COVID-19 infection. Most of the methods use CT scans, because CT scans provide more information than two-dimensional X-ray images. On the other hand, CT requires higher cost equipment and relatively higher radiation dose than CXR and may not be readily available in resource-limited communities. CXR is a widely used low-cost and efficient detection method that may be used in the evaluation of the degree of lung involvement at the initial screening of suspected cases of COVID-19 infection and subsequent monitoring over the course of treatment. Most importantly, CXR can be acquired with portable bedside equipment without moving the patient from isolated infectious disease wards to the CT suites.

Not all previous studies used imaging information. Feyaerts et al. [9] for instance proposed an integrated computational approach to analyze the combined plasma and single-cell proteomic data and the severity was classified as mild, moderate, or severe. The results provided a set of early determinants of COVID-19 severity that may point to therapeutic targets for the prevention of COVID19 progression, with the area under the receiver operating characteristic curve (AUC) ranging between 0.773 and 0.799. In a more recent work, Danilov et al. [10] proposed an approach for automatic scoring of COVID-19 severity by X-ray imaging based on a deep learning workflow. Their approach first segmented the lung and then scored the severity. Frid-Adar et al. [11] estimated the severity of pneumonia in COVID-19 patients. They segmented the lung and the region of pneumonia and then performed severity classification. Table 1 shows an overview of the related work. There is a limited number of published articles for detection of severe COVID-19 infection versus other severity classes as moderate, mild, and normal lungs. However, a larger amount of work was devoted to classifying COVID-19 infection versus normal lungs and/or pneumonia without considering its severity. The following works considered multi-class deep learning classification approaches for stratifying X-ray lung images into different number of categories. For instance, Namburu et al. [12] considered seven categories: COVID-19, virus, bacteria, ARDS, SARS, Streptococcus, and normal. Ren et al. [13] used four categories: COVID-19 infection, bacterial infection, virus infection, and normal lungs. Hadi et al. [14] considered only three categories: COVID-19, normal, and viral pneumonia, so did Constantinou et al. [15]: COVID-19, non-COVID-19, and normal, and Ullah et al. [16]: COVID-19, pneumonia, and normal. Some studies performed multi-region or multi-class classification of COVID-19 infection. Samala et al. [17] demonstrated that deep-learning-based quantitative severity descriptors on CXR images had significant correlation with radiologist’s severity ratings and had the potential to classify normal lungs from those with COVID-19 infection. Park et al. [18] divided the CXR images into six regions and the AI model analyzed each region to quantify the COVID-19 severity: 0 for normal and 1 for severe. In the end, COVID-19 severity was scored in a range between 0 and 6. Sahoo et al. [19] proposed a multi-stage system for COVID-19 severity assessment, classifying as mild, moderate, severe, and critical, on CXR images.

Although CXRs of COVID-19 are taken all around the world every day, available datasets for AI model training are still limited due to the lack of expert labels and the difficulties in sharing patient data outside the hospital for privacy issues [18]. There is a considerable number of datasets containing labels as negative or positive for COVID-19 [24,25,26]. For our work, we utilized the datasets with COVID-19 severity labels. To our knowledge, publicly available datasets of CXRs containing severity labels for COVID-19 are those shown in Table 2.

In this study, we classified the patient’s lung infection into severe vs. non-severe because severe COVID infection carries a higher risk for the patient and requires prompt treatment with oxygen therapy and hospitalization while less severe levels of infection often stay on observation. Also, severe infections are more likely to have long standing residual changes in their lungs and may need follow up imaging [33]. As summarized in Table 1, most of the previous studies that used CXRs classified COVID-19 versus non-COVID-19 rather than severe versus non-severe infection.

The specific contributions of our work include:

(1) Tackling a challenging task of classifying severe from non-severe lung infection in COVID patients using a dataset of CXR images containing various degrees of severity (normal, mild, moderate, and severe) infection.
(2) Studying the reproducibility of the performances of the deep learning models trained with data sets of limited sizes and from multi-institutional sources.
(3) Evaluating the generalizability of the trained deep learning models by using both independent internal and external test sets.

## 2. Materials and Methods

### 2.1. Datasets

We selected four databases in our study where images and/or cases contained severity labels as shown in Table 3. The selected databases were COVIDGR [23,31], BrixIA [28,29,30], MIDRC [32], and our institutional database from the University of Michigan (UMICH). Institutional Review Board (IRB) approval was obtained for retrospective collection of the UMICH database with waiver of informed consent. We did not include the data set by Cohen et al. [27] because their definitions of the levels of severity were very different from those of the other data sets. The combined dataset consisted of 5748 cases and 6193 CXR images with severity ratings as described in Table 2.

For the segmentation step, we used an ImageNet pre-trained U-Net model and further pre-trained it with a subset of the NIH CXR images (N = 277). The U-Net model was then fine-tuned and evaluated with a small subset of the MIDRC data set, including 30 images for training, 7 images for validation, and 39 images for testing. The reference segmentation of the lungs in these development data sets was manually outlined by trained researchers.

For our severity assessment study, we considered the images that had been labeled as one of the following: negative, mild, moderate, or severe defined by TCIA [7,8]:

1. Negative: negative for pneumonia, no lung opacities.
2. Mild: Required if not negative for pneumonia. Opacities in 1–2 lung zones.
3. Moderate: Required if not negative for pneumonia. Opacities in 3–4 lung zones.
4. Severe: Required if not negative for pneumonia. Opacities in >4 lung zones.

The MIDRC set included images in DICOM format. Images were acquired with flat-panel digital detectors (DX) and computed radiography (CR). Each image was read, and the COVID-19 severity was rated independently by three physicians. Most of the images had consistent ratings from the three readers. For the images that obtained different severity ratings, we used the majority (in case of 2 equal ratings) or average (in case of 3 different ratings) of the severity ratings as the label. The BrixIA set was contributed by an Italian group and included CXR images of COVID-19 subjects acquired with both CR and DX modalities. All data were anonymized DICOM files and annotation files in CSV with BrixIA score and relevant metadata. The severity ratings had consensus from five radiologists [30]. The COVIDGR set included anonymized X-ray images in Joint Photographic Experts Group (JPG) format. It was collected under a collaboration with an expert radiologist team of the Hospital Universitario San Cecilio, Spain [31]. The UMICH set included de-identified DX images in DICOM format. One experienced chest radiologist provided the severity rating according to the TCIA definition. A DICOM reader was used to read the CXR images in DICOM format that provides all information about the image in the header including the image size and gray level depth. The header information was used to read out the image as it was originally stored. The images were preprocessed as described in “Section 2.2.2.1. Data Harmonization”.

We proposed to develop a deep learning model that would classify severe lung infection versus the rest (normal, mild, and moderate) in COVID-19 patients. Table 3 shows the partitioning of the data sets by case into the training, validation, and test subsets, i.e., all images from the same patient were always grouped into the same subset to maintain the independence among the three subsets. It also shows the distribution of severe and non-severe images for each set. For training deep learning models, it is preferable to have a balanced dataset. However, severe and non-severe cases often are not proportionally distributed. Because of the limited sizes of the available datasets, we used all eligible images for this study.

### 2.2. Deep-Learning-Based Processing Pipeline

**Figure 2:**
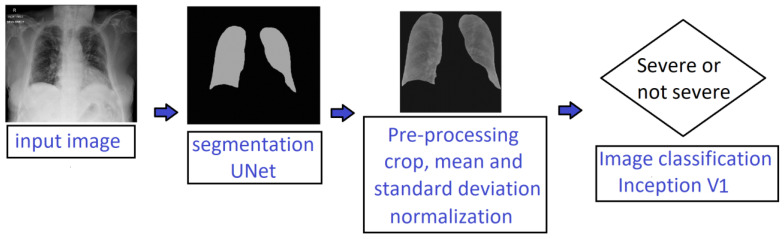
Processing pipeline for classification of severe versus non-severe COVID-19 patients using chest radiographs.

In this work, we implemented a deep-learning-based pipeline for automatically identifying severe lung infection on CXR images of COVID-19 patients. Figure 2 illustrates our proposed processing pipeline that consisted of two main stages: (1) lung segmentation and (2) severity classification. It combined deep learning models and conventional methods such as edge detection, hole filling, and basic statistical operations. Each stage is described in detail in the following subsections.

#### 2.2.1. Lung Segmentation

**Figure 3:**
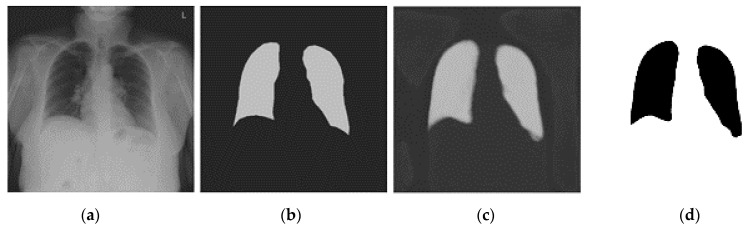
Lung Segmentation by U-Net: (**a**) Input image, (**b**) manually outlined lungs—the target output, (**c**) U-Net prediction and (**d**) final lung regions.

Figure 3 shows the process of our segmentation method. The segmentation was intended to focus the AI model on the regions within the lungs and avoid shortcut learning. We used U-Net [34] based architecture for lung segmentation.

The U-Net was trained using transfer learning with two pre-training stages: first by ImageNet, then by 277 CXR images of the NIH database [35], and finally fine-tuned by a small subset of the MIDRC data set. The pre-training with the NIH dataset adapted the weights to CXR images in general while the fine tuning with the MIDRC data set further adapted the U-Net to CXRs of COVID patients, thus improving the segmentation accuracy. The U-Net was trained to output the segmented right and left lungs (Figure 3). The U-Net segmented output image was thresholded and dilated to obtain the final binary lung mask. However, sometimes the U-Net output noise or disconnected parts of the lungs. For these situations, an automated post-processing technique was used to select and keep only the two biggest objects which were usually the lungs. Finally, a hole-filling technique was used to remove the holes, if any, in the segmented lung masks.

#### 2.2.2. Severity Classification

We trained three models by using different training datasets:

1. Model M: Trained only with the MIDRC training set.
2. Model MB: Trained with the combined MIDRC + BrixIA training set.
3. Model MBC: Trained with the combined MIDRC + BrixIA + COVIDGR training set.

For severity assessment, we used the Inception-v1 [36] and Inception-v4 [37] deep neural network architectures. The selection of the Inception architectures was based on our prior studies related to breast cancer diagnosis on mammograms and breast tomosynthesis images [38,39,40], where these architectures showed robust and accurate performance. Inception-v1 includes 9 inception blocks and 5 million parameters. Inception-v4 is a deeper architecture with 14 inception blocks and 43 million parameters and potentially has better learning capacity. Because of the limited training set sizes and training efficiency, the Inception-v1 structure was used in most of the experiments. Both models were pre-trained with ImageNet dataset. We fine-tuned the last 3 blocks of Inception-v1 and the last 2 blocks of Inception-v4. We also compared the performances of the Inception-v1 model with the Inception-v4 model.

The segmented lung regions from the U-Net were cropped with a square bounding box. The cropped region was resampled to a matrix size of 480 × 480 pixels. The segmented lung images focused the attention of the Inception network on only relevant information within the lungs by excluding the anatomical background structures.

##### 2.2.2.1. Data Harmonization

**Figure 4:**
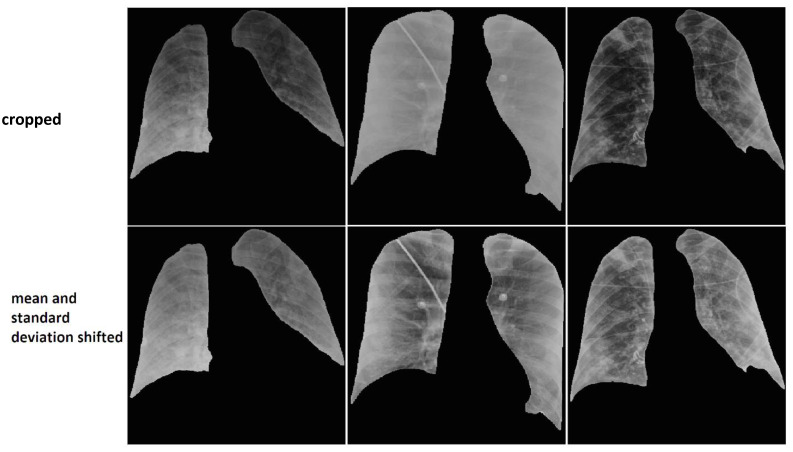
Original images are presented on the top row. Bottom images are harmonized by shifting the mean and the standard deviation of the pixel intensities within the lung regions. The background pixel intensity outside the lung region was set to a constant value of zero for all images.

To harmonize the wide variations of pixel intensity distributions of the CXR images from different equipment and clinical sites, we experimentally designed the following image pre-processing steps and selected the parameters using small subsets of the training and validation sets that were not part of the independent test set. We pre-processed the masked lung images by reducing the gray levels of all images to 8 bits, shifting the mean pixel intensity in the lung regions to 128, and scaling the standard deviation of all images to one single value, 22 for DX images and 26 for CR images. These values (22 and 26) were the average of the standard deviations of the pixel intensity distributions over images of the respective modality from the training set. The pixels outside the lung regions were set to a constant value of zero. Figure 4 presents six images where the images on the top row do not have the mean and standard deviation shifted and the images on the bottom row are processed by adjusting the mean and standard deviation. It can be observed that the images on the bottom row have more uniform appearance compared to the images on the top row.

##### 2.2.2.2. Data Augmentation

We applied data augmentation to the training data set where each image was flipped horizontally. The augmented data set was used as input to train the deep learning models.

Our experiments were performed on GPUs—Nvidia GeForce GTX 1080Ti with 11 GB of memory (Santa Clara, CA, USA).

## 3. Results

### 3.1. Lung Segmentation

The lung segmentation performance was assessed by comparing the U-Net model’s segmentation results to the manual outlines on the MIDRC test subset of 39 images. To assess the segmentation accuracy, we calculated the quantitative measures including the Jaccard index, the Dice Coefficient, the Hausdorff distance, and the average Euclidean distance relative to the reference. Table 4 shows the four mean segmentation performance measures obtained by averaging over the images in the test subset.

Although the lungs in general are correctly segmented, the lung segmentation on CR images is less accurate than on DX images. This is because the U-Net was trained with only DX images due to DX is the predominant modality and the manual outlines were obtained only for DX images.

**Figure 5:**
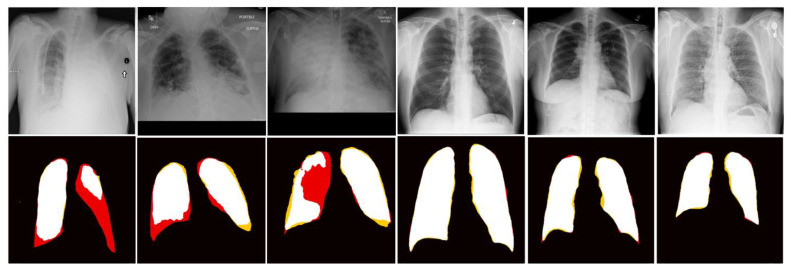
Images in the top row are input and images in the bottom row are segmentation results. Color labels: Black—background, white—labeled lung, red—false negative, and yellow—false positive.

Figure 5 illustrates six segmentation results on test images.

The three images on the left side demonstrate the limitation of the segmentation method where the lung region was almost invisible due to infection. The other three cases represent images for which the segmentation agrees well with the manual outlines.

### 3.2. Severity Assessment

**Figure 6:**
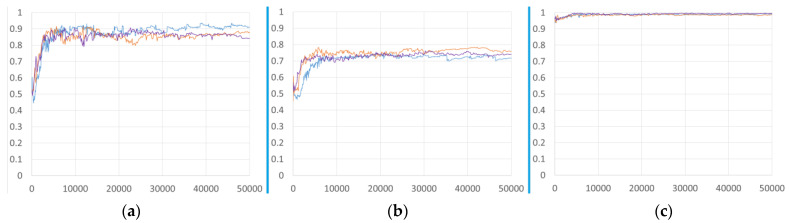
AUC-versus-iteration performance of Inception-v1 models trained with different training sets: Model M (blue line), Model MB (orange line), and Model MBC (purple line). The trained models were deployed on different validation sets: (**a**) MIDRC validation, (**b**) BrixIA validation, and (**c**) COVIDGR validation. The horizontal axis was plotted as the number of iterations because the number of images in each training set was very different.

To study the effects of different training sets on the performance of the deep learning model for severity classification, we trained the Inception-v1 model with three different training sets, described above. Figure 6 shows the AUC of three different models, M, MB, and MBC on three different validation sets as the number of iterations increased during training. There was no notable improvement with the addition of more data by combining the different data sets.

**Figure 7:**
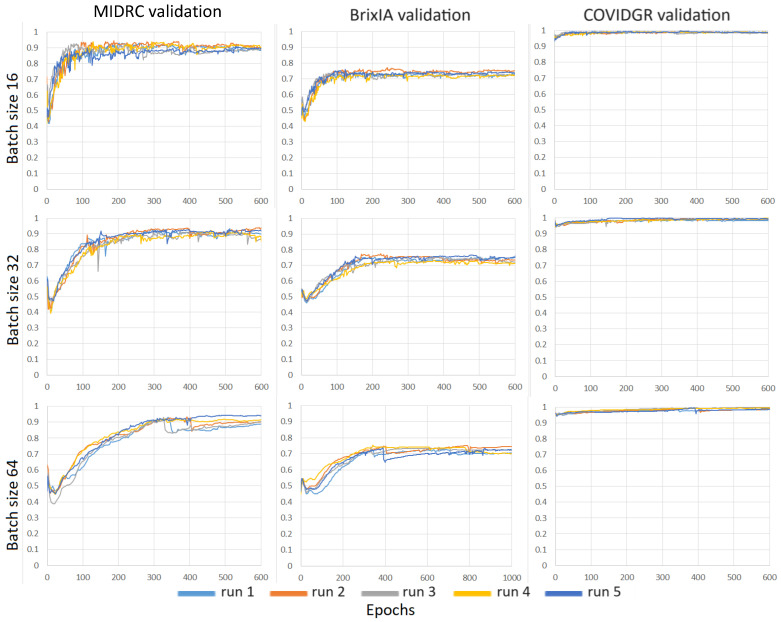
AUC-versus-epoch curves of Inception-v1 models trained on the MIDRC training set using different batch sizes (16, 32, and 64) and deployed on three different validation sets. Each model was trained with five different random initializations.

**Figure 8:**
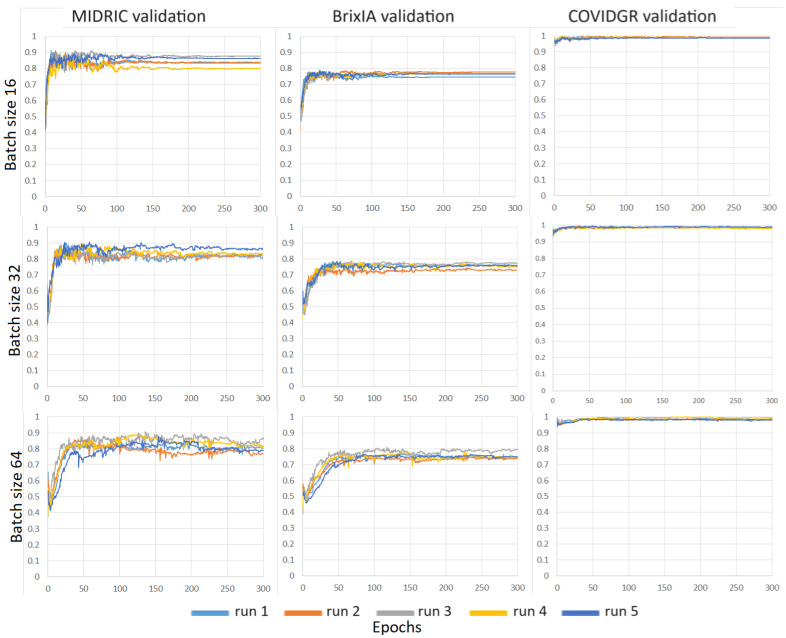
AUC-versus-epoch curves of Inception-v1 models trained on the combined MIDRC + BrixIA training set using different batch sizes (16, 32, and 64) and deployed on three different validation sets. Each model was trained with five different random initializations.

We evaluated the consistency of the models when we repeated the training with different random initialization. For each repeated experiment, the weights of the last fully connected layer were randomly initialized and the training dataset batches were sampled with a different random seed. We studied the impact of batch size on models trained on the MIDRC training dataset. Figure 7 shows the validation AUC-vs-epoch curves for batch sizes of 16, 32, and 64 for the Inception-v1 model trained with the MIDRC training set. We plotted the horizontal axis in terms of epoch because the number of epochs was kept the same for the training of the different models while the number of iterations scaled with the batch size (i.e., number of iterations = total number of training images/batch size). For each batch size, the model was trained with five different random initializations and deployed on the three different validation sets (MIDRC, BrixIA, and COVIDGR). The results show that the batch size of 16 obtained a more stable performance on the validation sets. The same trend can also be seen in Figure 8 where the models were trained with the combined MIDRC + BrixIA training set. The batch size of 16 was therefore used in most of our model training unless specified.

**Figure 9:**
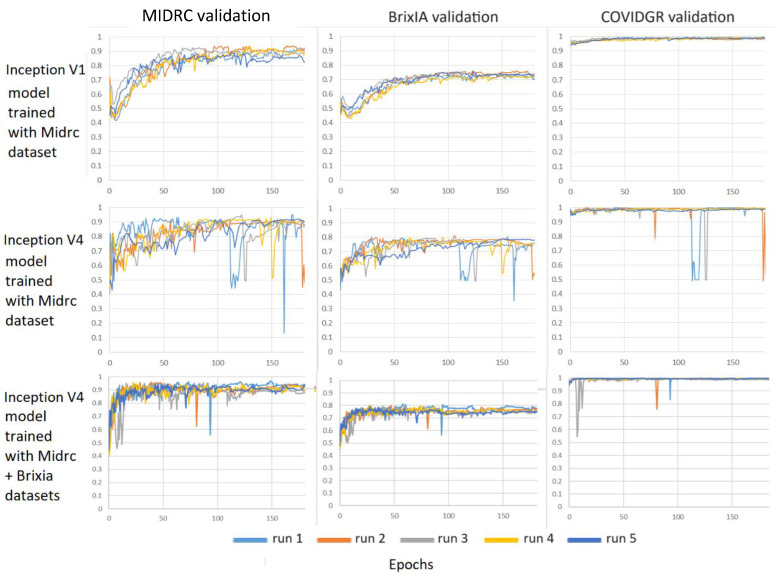
Comparison of the AUC-versus-epoch curves of Inception-v1 and Inception-v4 models trained on the MIDRC training set and the combined MIDRC + BrixIA training set and deployed on three different validation sets. Each model was trained with five different random initializations. Batch size = 16.

Figure 9 compares the performance of two different deep learning architectures, Inception-v1 and Inception-v4, on three different validation sets. The Inception-v1 model achieved better and more stable performance than the Inception-v4 model on the three different validation sets when they were both trained with the MIDRC training set. With the combined MIDRC + BrixIA training set, the Inception-v4 model could also converge to stable performance after about 150 epochs, indicating that the MIDRC training set may be too small to train the Inception-v4 model.

### 3.3. Severity Classification

After the models were trained, we selected a checkpoint as a frozen model in the relatively stable region of the validation curves and deployed the models to the independent held-out test sets. From the validation curves, we observed that 200 epochs could reach stability for the different conditions that we studied so that it was selected as the end point for all models. For each training set and batch size, we trained the model five times with different random initialization to perform sensitivity analysis and estimated the mean and standard deviation of the AUC on our deep learning pipeline for severity assessment. Table 5 shows the mean and the standard deviation of AUC deployed on different test sets for the Inception-v1 models trained with different training sets and batch sizes.

Similar results are shown in Table 6 for the Inception-v4 model except that it was trained only at batch sizes of 16 and 32. The Inception-v4 model with batch size of 64 could not be trained because of the limited memory size (11 GB) of our graphic processing units.

In addition to AUC, we also estimated the corresponding accuracy, sensitivity, and specificity at a cut-off point on the ROC curve determined by the maximum Youden Index for all the conditions reported in Table 5 and Table 6. These results are included in the Appendix A in Table A1, Table A2, Table A3 and Table A4.

## 4. Discussion

Deep-learning-based models have been proposed for detection of COVID-19 [41]. However, few studies were conducted for classification of severe versus non-severe lung infection on chest radiographs of COVID-19 patients using deep learning. Chest radiography is low cost and easily accessible even in resource-limited regions, especially that sequential imaging, including a reference baseline exam, is often needed for surveillance of infection progression or regression in many COVID patients. A deep learning model that can assist physicians in consistently identifying severe lung infection in COVID-19 patients would be useful for treatment management, especially in pandemic situations.

We demonstrated that it is possible to train the Inception-v1 and Inception-v4 models using the limited data sets with proper severity labels. For Inception-v1, the small MIDRC training set appeared to be sufficient and the addition of the other training sets did not substantially improve its performance. On the other hand, for Inception-v4, the model trained with the small MIDRC set was unstable likely because Inception-v4 had a much larger number of weights to be trained than Inception-v1. The larger BrixIA training set was needed to stabilize the training. The Inception-v4 model could achieve better performance than Inception-v1 when it was trained with the larger training set. This is consistent with the expectation that the larger Inception-v4 model has larger learning capacity but requires larger training sample size to learn properly.

The training sets for the deep learning models in this study were obtained from three public data sets, MIDRC, BrixIA, and COVIDGR, which were collected from different populations and different imaging equipment. The BrixIA and the COVIDGR sets were used in combination with the MIDRC set, in comparison to the MIDRC set alone. The independent test results in Table 5, Table 6, Table A1, Table A2, Table A3 and Table A4 showed a similar trend that the test performance of the trained model increased slightly when the training set size increased. For a given test set, the AUC increased when the training samples from the same population as the test set were included in the combined training set. The COVIDGR set appeared to be somewhat distinct from the other two training sets such that adding the COVIDGR set would change the test performance for the MIDRC and BrixIA test sets in both directions. One possible cause is that the COVIDGR set was provided in JPG format, which might change the image quality compared to the images in DICOM format.

Because of the limited size of each data set, we allocated only a small portion of the data set for validation. As can be seen from the validation curves, the AUC performance was quite different from the final test results. However, the small validation sets served adequately for the purpose of monitoring the convergence of the training procedures, indicating whether the training reached stability and eventually converged to a plateau region under the various training conditions. The test results were much more consistent among the different training conditions and test sets.

The UMICH test set was collected from a different institution than the training or validation sets. It served as an “external validation set” in this study. The results indicated that the trained deep learning models can be generalized to an unseen dataset from a different population.

It is difficult to make a direct comparison between the performance of our models and the previous studies (Table 1). The performance of a model depends strongly on the characteristics of the data set and the reference standard. The previous studies used different data sets than those in our study. It is also unclear if any of the studies that used CXRs as input targeted the same task, i.e., classifying severe vs. non-severe lung infection in COVID-19 patients since the definition of the severity levels in the different studies appeared very different. Ref. [23] might be the closest but it did not report AUC; the accuracy, sensitivity, and specificity were comparable to our values. Regardless of the details of the studies, most of the reported AUCs were lower than those achieved by our models.

There are limitations in this study. The sizes of all four data sets were small. One of the challenges is that most of the publicly available data sets did not use the same severity ratings defined by TCIA, probably because they require experienced chest radiologists’ reading. The MIDRC data set has been enlarged substantially since the early set that we used for this study. However, different methods are used to assess the severity of COVID-19 infection for the newly added chest radiographs so that the labels are inconsistent with the early set and cannot be combined together for our purpose. This therefore reveals an important issue in the collection of large database such as MIDRC; it is preferable to plan from the beginning the methods and standards for collecting the labels and annotations required for the various applications of the data so that they can be more uniform for the entire data set. The changes in labeling or associated information from time to time would fragment the database into smaller subsets that contain consistent information for a given study, partly defeating the purpose of collecting large public database.

A second limitation is that the image characteristics of the public data sets were heterogeneous. The MIDRC set included images acquired with CR, for which the image quality and the processing methods were very different from images acquired with flat-panel digital detectors. The images in the COVIDGR set were stored in JPG format, different from the DICOM standard format for medical images. The heterogeneity of the image characteristics might have reduced the effectiveness of combining the training sets together to increase the training set sizes. As seen in Table 5 and Table 6, there were only relatively small improvements in the test AUCs when the BrixIA training set, which was several times larger, was added to the MIDRC training set. On the other hand, there may be an advantage that the model trained with such heterogeneous data may be more robust when it is deployed to local patient images of different quality, also apparent from the tables showing relatively consistent performances across the different independent test sets from multi-institutions and different countries.

A third limitation is that we did not compare many different deep learning structures, including the more recent visual transformer approach. However, the purpose of this study is to evaluate the feasibility of training a robust model despite the limited sample size and heterogeneous quality of the available data, which would likely be the more important factors that determined the model performance than the deep learning architectures.

## 5. Conclusions

In this study we developed a deep learning pipeline to differentiate severe from non-severe lung infection in COVID-19 patients using chest radiographs. A U-Net model was developed to segment the lungs. Inception-v1 and Inception-v4 models were trained for the classification of severe vs. non-severe lung infection. Multi-institutional datasets were used to develop and evaluate the models. Different combinations of training and validation data sets were used to study the reproducibility of classification performance. The generalizability of the trained deep learning models was validated with multi-institutional test sets and an external test set. The Inception-v1 models achieved AUCs ranging from 0.81 ± 0.02 to 0.84 ± 0.0, while the Inception-v4 models achieved AUCs in the range between 0.85 ± 0.06 and 0.89 ± 0.01 on the independent test sets. Our study shows promise in differentiating COVID-19 patients with severe from non-severe lung infection on chest radiographs. Future work should improve the performance of the deep learning models by increasing the training sample sizes, if available. COVID-19 is evolving over time and vaccination also changes the course of the viral infection manifestation and symptoms. It is hoped that these early works can serve as a foundation for continued development of updated image analysis tools that can assist in early and efficient detection of the disease and thus facilitate proper treatment decision.

## Figures and Tables

**Table 1:** Related work on classification of COVID-19 severity or from other lung infections.

Method	Year	Data	Class Groups	Results
Statistical approach [9]	2021	Plasma and single-cell proteomic	Mild, moderate, severe	AUC_training_ = 0.799 AUC_validation_ = 0.773
Deep Learning [10]	2022	CXR	COVID-19 and normal	Mean absolute error of 0.30
Deep Learning [11]	2021	CXR	Severity level (scores from 0 to 8)	confusion matrix: Sensitivity = 0.94 Specificity = 0.98
Deep learning [12] ResNet	2022	CXR	COVID-19, virus, bacteria, ARDS, SARS, Streptococcus, normal	Accuracy = 98%
Deep learning [13]	2023	CXR	COVID-19, virus, bacteria, and normal	Accuracy = 97.65%
Deep learning [14] CORONA-NET	2023	CXR	COVID-19, viral pneumonia, normal	Accuracy = 99.57%
Deep learning [15] ResNet	2023	CXR	COVID-19, non-COVID-19, and normal	Accuracy = 96%
Deep learning [16] DAM-Net	2023	CXR	COVID-19, pneumonia, and normal	Accuracy = 97.22% Sensitivity = 96.87% Specificity = 99.12%
Deep learning [17]	2021	CXR	Severity descriptors vs. radiologist’s severity ratings, normal vs. abnormal	Correlation = 0.68 (*p* < 0.0001) AUC = 0.78
Cytokine profiles with statistical approaches [20]	2022	Cytokine concentration	Severe, moderate, and mild	AUC = 0.83
Supervised machine learning models [21]	2023	Feature-dataset consisting of the routine blood values and demographic data that affect the prognosis of COVID-19	Severely and mildly	AUC range: 0.75 to 0.95 Accuracy range: 94.05% to 97.86%
Vision Transformers [22]	2022	CXR and CT	COVID-19, pneumonia, and normal	Accuracy = 94.62%
COVID_SDNet [23]	2020	CXR	Severe, moderate, and mild	Accuracy = 81.0% ± 2.9% Sensitivity = 76.8% ± 6.3% Specificity = 85.2% ± 5.4%
Multi-task vision [18]	2022	CXR	Severity degree from 0 to 6	Accuracy range: 78.7% to 97.7%
Multi-stage framework [19]	2023	CXR	Mild, moderate, severe, and critical	Accuracy = 97.63%

**Table 2:** Available databases for COVID-19 (public data sets with severity rating labels available for the current study).

Name of Database	Year of Data Collection	Type of Labels	Public Available	Severity COVID19 Rate	Number of Images
Cohen et al. [27]	2020	Defined by other paper [9]: if “survival” is false, it is called “high severity”; if “survival” and “went-ICU” are true, it is termed “moderate severity”; if “survival” is true and “went-ICU” is false, it is named “low severity”	yes	yes	679 X-ray images from 412 patients
BrixIA [28,29,30]	2020	The lungs were divided into six regions and each region was scored ranging from 0 to 3. Consensus from 5 radiologists	yes	yes	4695
COVIDGR [23,31]	2020	Data were rated by 1 radiologist as negative, mild, moderate, or severe	yes	yes	852
MIDRC [32]	2022	Data were rated by 3 radiologists as negative, mild, moderate, or severe	yes	yes	823 with severity labels for COVID-19

**Table 3:** Data collection. Distribution of severe and non-severe images and cases per training, validation, and testing dataset.

		TRAINING			VALIDATION			TESTING		
		Total	Severe	Non-Severe	Total	Severe	Non-Severe	Total	Severe	Non-Severe
MIDRC	images	582	129 (22%)	453 (78%)	68	18 (26%)	50 (74%)	173	70 (40%)	103 (60%)
	cases	425			50			26		
BrixIA	images	3285	563 (17%)	2722 (83%)	470	58 (12%)	412 (88%)	940	184 (20%)	756 (80%)
	cases	3206			464			917		
COVIDGR	images	300	56 (19%)	244 (81%)	42	7 (17%)	35 (83%)	83	15 (18%)	68 (82%)
	cases	300			42			83		
UMICH	images	--	--	--	--	--	--	250	86 (34%)	164 (66%)
	cases	--			--			235		

**Table 4:** The performance measures for lung segmentation in the MIDRC test set using the U-Net deep learning approach.

	Mean of Jaccard	Mean of Dice Coefficient	Mean of Hausdorff Distance	Mean of Average Euclidean Distance
Mean	0.82	0.90	37.7 mm	5.4 mm
Standard deviation	0.07	0.04	24.6 mm	2.7 mm

**Table 5:** AUC values for classification of severe and non-severe COVID-19 based on chest radiographs in the held-out independent test sets by the Inception-v1 models trained with different training sets and batch sizes of 16, 32, and 64. The mean and standard deviation were estimated from the models trained with five random initializations for a given training set and batch size.

Batch Size	Models	MIDRC	BrixIA	COVIDGR	UMICH
		AUC			
16	*M*	0.82 ± 0.01	0.82 ± 0.01	0.80 ± 0.04	0.80 ± 0.01
	*MB*	0.84 ± 0.01	0.84 ± 0.01	0.78 ± 0.02	0.80 ± 0.02
	*MBC*	0.84 ± 0.02	0.85 ± 0.00	0.82 ± 0.02	0.80 ± 0.01
32	*M*	0.82 ± 0.02	0.81 ± 0.01	0.75 ± 0.04	0.77 ± 0.01
	*MB*	0.82 ± 0.02	0.84 ± 0.01	0.76 ± 0.03	0.80 ± 0.01
	*MBC*	0.83 ± 0.02	0.84 ± 0.00	0.84 ± 0.01	0.81 ± 0.02
64	*M*	0.79 ± 0.03	0.76 ± 0.02	0.62 ± 0.07	0.71 ± 0.05
	*MB*	0.83 ± 0.02	0.83 ± 0.01	0.78 ± 0.06	0.78 ± 0.02
	*MBC*	0.82 ± 0.03	0.83 ± 0.01	0.77 ± 0.03	0.78 ± 0.02

**Table 6:** AUC values for classification of severe and non-severe COVID-19 based on chest radiographs in the held-out independent test sets by the Inception-v4 models trained with different training sets and batch sizes of 16 and 32. The mean and standard deviation were estimated from the models trained with five random initializations for a given training set and batch size.

Batch Size	Models	MIDRC	BrixIA	COVIDGR	UMICH
		AUC			
16	*M*	0.82 ± 0.09	0.79 ± 0.14	0.82 ± 0.15	0.82 ± 0.10
	*MB*	0.86 ± 0.01	0.88 ± 0.01	0.79 ± 0.04	0.89 ± 0.01
	*MBC*	0.88 ± 0.01	0.87 ± 0.01	0.84 ± 0.02	0.89 ± 0.01
32	*M*	0.84 ± 0.03	0.85 ± 0.01	0.74 ± 0.05	0.87 ± 0.02
	*MB*	0.88 ± 0.02	0.88 ± 0.01	0.79 ± 0.03	0.89 ± 0.02
	*MBC*	0.87 ± 0.01	0.88 ± 0.01	0.85 ± 0.06	0.89 ± 0.01

## Data Availability

Data available upon request.

## Appendix A

We also have estimated the accuracy, sensitivity, and specificity for all the experiments in Table 5 and Table 6. The results are presented in Table A1, Table A2, Table A3 and Table A4 below. The accuracy, sensitivity, and specificity were estimated at a cut-off point on the ROC curve determined by the maximum Youden Index.

**Table A1:** Accuracy values for classification of severe and non-severe COVID-19 based on chest radiographs in the held-out independent test sets by the Inception-v1 models trained with different training sets and batch sizes of 16, 32, and 64. The mean and standard deviation were estimated from the models trained with five random initializations for a given training set and batch size.

Batch Size	Models	MIDRC	BrixIA	COVIDGR	UMICH
		Accuracy			
16	*M*	0.76 ± 0.01	0.74 ± 0.01	0.74 ± 0.04	0.73 ± 0.01
	*MB*	0.77 ± 0.01	0.77 ± 0.02	0.72 ± 0.03	0.75 ± 0.02
	*MBC*	0.77 ± 0.02	0.77 ± 0.01	0.76 ± 0.06	0.73 ± 0.02
32	*M*	0.75 ± 0.02	0.75 ± 0.02	0.71 ± 0.05	0.73 ± 0.01
	*MB*	0.75 ± 0.03	0.77 ± 0.02	0.76 ± 0.06	0.75 ± 0.02
	*MBC*	0.78 ± 0.01	0.78 ± 0.01	0.76 ± 0.04	0.75 ± 0.02
64	*M*	0.74 ± 0.04	0.73 ± 0.03	0.66 ± 0.07	0.68 ± 0.04
	*MB*	0.77 ± 0.03	0.78 ± 0.02	0.76 ± 0.10	0.74 ± 0.02
	*MBC*	0.75 ± 0.03	0.75 ± 0.01	0.74 ± 0.03	0.73 ± 0.02

**Table A2:** Accuracy values for classification of severe and non-severe COVID-19 based on chest radiographs in the held-out independent test sets by the Inception-v4 models trained with different training sets and batch sizes of 16 and 32. The mean and standard deviation were estimated from the models trained with five random initializations for a given training set and batch size.

Batch Size	Models	MIDRC	BrixIA	COVIDGR	UMICH
		Accuracy			
16	*M*	0.77 ± 0.05	0.75 ± 0.08	0.82 ± 0.05	0.77 ± 0.06
	*MB*	0.80 ± 0.01	0.81 ± 0.02	0.74 ± 0.05	0.84 ± 0.01
	*MBC*	0.80 ± 0.02	0.79 ± 0.02	0.81 ± 0.05	0.83 ± 0.02
32	*M*	0.77 ± 0.03	0.78 ± 0.02	0.71 ± 0.07	0.80 ± 0.02
	*MB*	0.81 ± 0.02	0.79 ± 0.02	0.71 ± 0.02	0.83 ± 0.01
	*MBC*	0.80 ± 0.01	0.79 ± 0.01	0.80 ± 0.07	0.83 ± 0.03

**Table A3:** Sensitivity and specificity values for classification of severe and non-severe COVID-19 based on chest radiographs in the held-out independent test sets by the Inception-v1 models trained with different training sets and batch sizes of 16, 32, and 64. The mean and standard deviation were estimated from the models trained with five random initializations for a given training set and batch size.

Batch Size	Models	Performance Metric	MIDRC	BrixIA	COVIDGR	UMICH
16	*M*	Sensitivity	0.75 ± 0.06	0.77 ± 0.02	0.77 ± 0.04	0.77 ± 0.05
		Specificity	0.77 ± 0.05	0.73 ± 0.02	0.74 ± 0.04	0.72 ± 0.03
	*MB*	Sensitivity	0.77 ± 0.04	0.76 ± 0.04	0.75 ± 0.11	0.71 ± 0.02
		Specificity	0.78 ± 0.04	0.77 ± 0.03	0.71 ± 0.06	0.77 ± 0.02
	*MBC*	Sensitivity	0.78 ± 0.03	0.79 ± 0.01	0.76 ± 0.09	0.77 ± 0.03
		Specificity	0.76 ± 0.05	0.77 ± 0.01	0.76 ± 0.08	0.71 ± 0.03
32	*M*	Sensitivity	0.78 ± 0.03	0.75 ± 0.05	0.68 ± 0.06	0.71 ± 0.05
		Specificity	0.73 ± 0.03	0.75 ± 0.04	0.72 ± 0.07	0.73 ± 0.02
	*MB*	Sensitivity	0.76 ± 0.06	0.76 ± 0.04	0.69 ± 0.12	0.71 ± 0.02
		Specificity	0.74 ± 0.08	0.78 ± 0.04	0.78 ± 0.09	0.77 ± 0.02
	*MBC*	Sensitivity	0.79 ± 0.04	0.76 ± 0.02	0.84 ± 0.04	0.73 ± 0.05
		Specificity	0.77 ± 0.03	0.78 ± 0.02	0.74 ± 0.06	0.76 ± 0.05
64	*M*	Sensitivity	0.75 ± 0.04	0.66 ± 0.05	0.67 ± 0.11	0.66 ± 0.08
		Specificity	0.73 ± 0.04	0.75 ± 0.05	0.66 ± 0.07	0.69 ± 0.06
	*MB*	Sensitivity	0.78 ± 0.03	0.75 ± 0.04	0.67 ± 0.11	0.69 ± 0.05
		Specificity	0.77 ± 0.04	0.79 ± 0.03	0.79 ± 0.15	0.77 ± 0.03
	*MBC*	Sensitivity	0.77 ± 0.03	0.78 ± 0.02	0.69 ± 0.08	0.69 ± 0.02
		Specificity	0.74 ± 0.06	0.74 ± 0.02	0.76 ± 0.04	0.75 ± 0.02

**Table A4:** Sensitivity and specificity values for classification of severe and non-severe COVID-19 based on chest radiographs in the held-out independent test sets by the Inception-v4 models trained with different training sets and batch sizes of 16 and 32. The mean and standard deviation were estimated from the models trained with five random initializations for a given training set and batch size.

Batch Size	Models	Performance Metric	MIDRC	BrixIA	COVIDGR	UMICH
16	*M*	Sensitivity	0.75 ± 0.11	0.71 ± 0.15	0.80 ± 0.16	0.77 ± 0.12
		Specificity	0.79 ± 0.04	0.76 ± 0.06	0.82 ± 0.04	0.77 ± 0.04
	*MB*	Sensitivity	0.82 ± 0.04	0.78 ± 0.03	0.72 ± 0.09	0.82 ± 0.04
		Specificity	0.78 ± 0.04	0.81 ± 0.03	0.75 ± 0.07	0.85 ± 0.03
	*MBC*	Sensitivity	0.82 ± 0.05	0.78 ± 0.01	0.79 ± 0.03	0.81 ± 0.05
		Specificity	0.79 ± 0.04	0.79 ± 0.02	0.82 ± 0.06	0.84 ± 0.03
32	*M*	Sensitivity	0.77 ± 0.04	0.78 ± 0.04	0.69 ± 0.08	0.84 ± 0.02
		Specificity	0.77 ± 0.05	0.78 ± 0.04	0.71 ± 0.08	0.78 ± 0.02
	*MB*	Sensitivity	0.81 ± 0.02	0.79 ± 0.02	0.75 ± 0.06	0.84 ± 0.05
		Specificity	0.80 ± 0.03	0.79 ± 0.03	0.70 ± 0.03	0.83 ± 0.03
	*MBC*	Sensitivity	0.78 ± 0.04	0.81 ± 0.03	0.85 ± 0.09	0.83 ± 0.03
		Specificity	0.81 ± 0.03	0.79 ± 0.02	0.79 ± 0.10	0.83 ± 0.05
